# Re-evaluation of arterial dissection as a possible major cause of lateral medullary infarction in a single-center study from South Korea

**DOI:** 10.1038/s41598-025-11860-8

**Published:** 2025-07-19

**Authors:** Jiyeon Ha, Wookjin Yang, Eung-Joon Lee, Han-Yeong Jeong, Matthew Chung, Hyemin Jang, Jeong-Min Kim, Keun-Hwa Jung, Seung-Hoon Lee

**Affiliations:** 1 Department of Neurology, Seoul National University Hospital, Seoul National University College of Medicine, 101 Daehak-ro, Jongno-gu, Seoul, 03080 Republic of Korea; 2 Department of Neurology, Asan Medical Center, Seoul, Republic of Korea

**Keywords:** Dissection, Blood vessel [C14.907.055.448], Lateral medullary syndrome [C10.228.140.300.775.200.100.500], Stroke [C14.907.253.855], Neuroimaging [E01.370.376.537], Magnetic resonance imaging [E01.370.350.825.500], Diagnosis, Differential [E01.171], Stroke, Stroke, Diagnosis

## Abstract

Limited sensitivity of conventional MRI and the invasiveness of TFCA have restricted the diagnosis of arterial dissection in lateral medullary infarction (LMI), leading to its underestimation and obscuring its clinical significance. However, high-resolution vessel wall MRI (HR-VWMRI) now allows more accessible and accurate diagnosis of dissection, prompting reevaluation of its prevalence. This single-center retrospective study included the LMI patients, among whom a subset underwent advanced imaging as HR-VWMRI or TFCA to confirm definite arterial dissection, and identified clinical factors associated with dissection. A risk scoring system for dissection was then developed based on these factors, and possible dissection was defined using the optimal cutoff value. Among 87 LMI patients, 47 underwent advanced imaging, with 24 (51.1%) diagnosed with definite dissection. Younger age, lower body mass index, headache, and absence of hypertension were associated with definite dissection and constructed dissection score, which showed 87.5% sensitivity and 73.9% specificity. Among the remaining 38 without advanced imaging, 12 were classified as possible dissection. Definite and possible dissections collectively accounted for 43.7% of LMI, exceeding large-artery atherosclerosis. These findings suggest dissection may be a predominant cause of LMI, underscoring the need for heightened awareness in clinical practice and proactive, risk-stratified diagnosis of arterial dissection.

**Supplementary Information:**

The online version contains supplementary material available at 10.1038/s41598-025-11860-8.

## Introduction

The anatomical location of stroke frequently offers valuable insight into its underlying mechanism, making lesion-specific patterns an important diagnostic clue. Although lateral medullary infarction (LMI) is clinically distinguished by its characteristic neurological features, its etiology has received relatively little attention in practice. Since the initial reports on the etiology of LMI appeared several decades ago^1,2^, its epidemiologic profile has rarely been reexamined with contemporary high-resolution imaging modalities. Consequently, large‑artery atherosclerosis has continued to be regarded as the principal cause of LMI^3,4^, and in the absence of early strong evidence for alternative etiologies, clinicians may prematurely close the diagnostic process by attributing the infarction to atherosclerosis.

However, arterial dissection represents a critical yet frequently underrecognized etiology of LMI^1,2^. Given the vascular supply of lateral medulla, LMI is particularly likely to result from local vascular pathologies such as dissection. However, the prevalence of dissection in LMI has been reported to be less than 30%, ranging from 15 to 26.4%^1,5^. Nevertheless, these epidemiological estimates were derived from analyses based exclusively on conventional imaging modalities, such as time-of-flight magnetic resonance angiography (TOF-MRA) or computed tomography angiography (CTA), which have limited resolution and sensitivity^6^. With the introduction of high-resolution vessel wall MRI (HR-VWMRI), noninvasive detection of pathologies in small-caliber vessels, such as vertebral artery (VA) and posterior inferior cerebellar artery (PICA) has become feasible^2,6,7^, necessitating the re-evaluation of the etiological distribution of LMI. Although some recent studies have addressed the etiology of LMI^8–10^, they still did not incorporate data from advanced imaging modalities, limiting the reliability of their epidemiological estimates.

LMI tends to occur at a younger age and is less frequently associated with conventional vascular risk factors than other stroke subtypes^1,2,11,12^. These features have been reported to be associated with arterial dissection^7^, indicating that dissection may account for a larger proportion of LMI cases than currently recognized. Nevertheless, limited clinical attention to dissection in LMI, combined with reliance on individual clinicians’ subjective judgment of each predictor rather than a structured risk assessment approach, may have led to its underestimation.

The present study therefore reassessed the prevalence of arterial dissection in LMI using data that incorporated advanced imaging, identified clinical factors associated with dissection, and developed a scoring system as an exemplary approach to guide risk stratification and proactive diagnostic assessment for dissection. This enabled a contemporary evaluation of the prevalence and clinical significance of arterial dissection in LMI.

## Methods

### Study population

Seoul National University Hospital (SNUH) Stroke Registry is a single center prospective database that collects information on consecutive patients admitted to SNUH for acute stroke within 7 days of symptom onset. SNUH, located in the Seoul metropolitan area of South Korea, is a high-volume center with comprehensive stroke unit. From this registry, a retrospective analysis was conducted on consecutive patients with lateral medullary infarction (LMI) admitted between January 2010 and December 2021. Eligible patients were adults whose diffusion-weighted MRI demonstrated an acute infarct in the lateral medulla. Patients were excluded if imaging revealed any additional ischemic lesions outside the lateral medulla. Patients whose baseline brain MRI and intracranial MRA were insufficient of diagnostic quality, or who had incomplete clinical data were also excluded. The study conforms to the Declaration of Helsinki and was approved by the Institutional Review Board of Seoul National University Hospital (IRB No. 1009-062-332); the requirement for written informed consent was waived because of the retrospective design and use of fully anonymized data.

### Clinical information and imaging parameters

Data regarding sex, age, body mass index (BMI), hypertension, diabetes, hyperlipidemia, stroke history, and smoking status were collected from all patients. Information on clinical presentation, including the presence of headache (either simultaneous or preceding other neurological deficits), history of cervical trauma within 1 month of stroke, and neurological symptoms were also collected. Additionally, symptoms and signs prevalent in LMI—such as gait disturbance, dizziness/vertigo, sensory abnormality, lateropulsion, dysarthria, limb ataxia, nausea/vomiting, dysphagia, facial palsy, Horner syndrome, limb weakness, and hoarseness—were collected.

### Imaging analysis

Conventional brain MRI protocols included the acquisition of diffusion-weighted imaging (DWI), apparent diffusion coefficient (ADC), T1- and T2-weighted imaging, fluid-attenuated inversion recovery (FLAIR) images, susceptibility-weighted imaging (SWI), and 3D time-of-flight magnetic resonance angiography (3D TOF-MRA). The conventional brain MRI findings of the VA or PICA ipsilateral to the site of infarction were classified into four categories for all patients included in the study: (1) Normal (Fig. S1A), (2) vertebral artery hypoplasia, stenosis, or occlusion (Fig. S1B), (3) focal stenosis and dilatation (also known as pearl-and-string sign) of VA or PICA (Fig. S1C), which is not traditionally considered pathognomonic but is highly associated with intracranial dissection^2,7,13^, (4) pathognomonic findings of dissection (defined as having at least one of the following: dissecting aneurysm (Fig. S2A), intramural hematoma (Fig. S2B), intraluminal flap (Fig. S2C), or double-lumen appearance (Fig. S2D))^6,7,14^.

A subset of the study population underwent advanced imaging to evaluate the VA and PICA, with high-resolution vessel-wall MRI (HR-VWMRI) or transfemoral cerebral angiography (TFCA) performed at the clinician’s discretion when further etiological assessment was warranted. These advanced imaging techniques are recognized for their high performance in confirming arterial dissections^15^.

Definite dissection was diagnosed when at least one pathognomonic feature (dissecting aneurysm, intramural hematoma, intraluminal flap, or double-lumen appearance; Fig. S2)^6,13,14^ was identified on either conventional MRI or advanced imaging. Two experienced neurologists (JH and WY), blinded to all clinical information, independently reviewed the images; any disagreements were resolved by consensus with a senior investigator.

### Predictors of dissection and score derivation

**Fig. 1:**
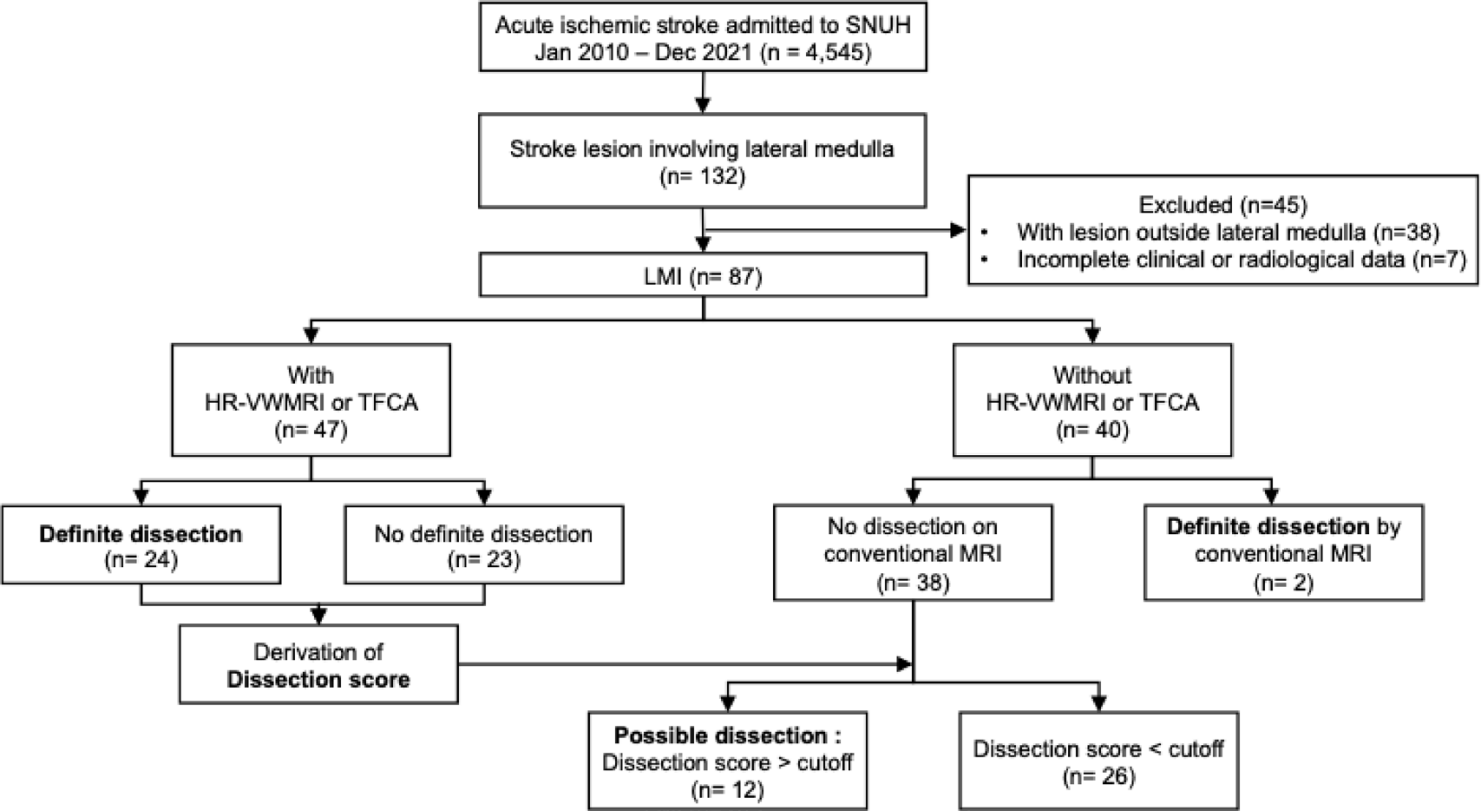
Flowchart of the study. SNUH, Seoul National University Hospital; LMI, Lateral medullary infarction; MRI, Magnetic resonance imaging; HR-VWMRI, High-resolution vessel wall MRI; TFCA, Transfemoral cerebral angiography.

Analyses were stratified based on the availability of advanced imaging (Fig. 1). Among individuals who underwent advanced imaging, baseline characteristics were compared between those with and without definite dissection. Clinical variables with a *P*-value < 0.10 were evaluated for their association with definite dissection using univariable and multivariable logistic regression, with definite dissection as the dependent variable.

The dissection score was derived by dividing each β coefficient from the multivariable model by the smallest absolute β and rounding to the nearest integer; the resulting integers were then assigned as point values for each covariate in the score^16^. Receiver operating characteristic (ROC) curve analysis was performed to demonstrate the predictive efficacy of the dissection score for identifying definite dissection. The optimal cutoff value was determined using the Youden index with the highest sensitivity and specificity.

### Definition of possible dissection

The dissection score was developed to estimate the risk of dissection in patients who neither underwent advanced imaging nor had definitive findings of dissection on conventional MRI. For these patients, a possible dissection case was defined as one with a dissection score above the optimal cutoff.

### Estimation of dissection in LMI

Based on the above definitions, LMI patients were classified into the following groups. Among those who underwent advanced imaging, cases were categorized as either definite dissection or no dissection. Among patients without advanced imaging, cases were classified as definite dissection (diagnosed by conventional MRI), possible dissection (dissection score above the cutoff), or low dissection score (below the cutoff). The proportions of each group were evaluated. For the total LMI cohort, the overall distribution of dissection and other etiologies was assessed, with both definite and possible dissection cases included in the dissection category.

### Statistical analysis

Continuous variables are expressed as mean ± standard deviation, and categorical variables as frequencies and percentages. Student’s *t*-test was used to compare continuous variables between groups, whereas the χ^2^ test or Fisher’s exact test was applied to categorical variables, as appropriate.

Statistical significance was indicated by *P* < 0.05. All analyses were performed using R version 4.1.3 (R Foundation for Statistical Computing, Vienna, Austria).

## Results

Of the 4,545 consecutive patients admitted to SNUH for acute stroke between January 2010 and December 2021, 132 had stroke lesions involving the lateral medulla. Of these, 38 patients were excluded due to concomitant lesions outside the lateral medulla, and 7 were excluded due to incomplete imaging or clinical data, leaving 87 patients with LMI for inclusion in the study (Fig. 1). The mean age of the included patients was 57.4 ± 15.4 years, and 63 (72.4%) were male. Eleven patients (12.6%) had pathognomonic findings of arterial dissection on conventional MRI (Table 1).

**Table 1:** Baseline characteristics by advanced imaging status.

	Total (n = 87)	HR-VWMRI/TFCA (n = 47)	No HR-VWMRI/TFCA (n = 40)	*P*-value
Age	57.4 ± 15.4	52.5 ± 15.2	63.3 ± 13.5	0.001
Sex, male	63 (72.4%)	34 (72.3%)	29 (72.5%)	> 0.99
BMI	24.7 ± 3.1	24.9 ± 3.3	24.5 ± 2.9	0.567
HT	56 (64.4%)	25 (53.2%)	31 (77.5%)	0.033
DM	26 (29.9%)	9 (19.1%)	17 (42.5%)	0.033
HL	58 (66.7%)	33 (70.2%)	25 (62.5%)	0.594
Smoking	45 (51.7%)	23 (48.9%)	22 (55.0%)	0.727
Stroke history	14 (16.1%)	3 (6.4%)	11 (27.5%)	0.017
Onset situation				> 0.99
During activity	62 (71.3%)	33 (70.2%)	29 (72.5%)	
Wake-up	25 (28.7%)	14 (29.8%)	11 (27.5%)	
Headache				0.003
Simultaneous	21 (24.1%)	8 (17.0%)	13 (32.5%)	
Preceding	24 (27.6%)	20 (42.6%)	4 (10.0%)	
Prior cervical trauma (n = 64)	27 (42.2%)	21 (55.3%)	6 (23.1%)	0.021
Gait disturbance	69 (79.3%)	35 (74.5%)	34 (85.0%)	0.346
Dizziness/vertigo	66 (75.9%)	35 (74.5%)	31 (77.5%)	0.938
Lateropulsion	66 (75.9%)	31 (66.0%)	35 (87.5%)	0.037
Sensory abnormality	61 (70.1%)	37 (78.7%)	24 (60.0%)	0.096
Dysarthria	44 (50.6%)	23 (48.9%)	21 (52.5%)	0.907
Limb ataxia	43 (49.4%)	20 (42.6%)	23 (57.5%)	0.24
Nausea/vomiting	39 (44.8%)	22 (46.8%)	17 (42.5%)	0.852
Dysphagia	37 (42.5%)	19 (40.4%)	18 (45.0%)	0.832
Facial palsy	30 (34.5%)	15 (31.9%)	15 (37.5%)	0.749
Horner syndrome	30 (34.5%)	16 (34.0%)	14 (35.0%)	> 0.99
Limb weakness	17 (19.5%)	14 (29.8%)	3 (7.5%)	0.019
Hoarseness	17 (19.5%)	9 (19.1%)	8 (20.0%)	> 0.99
Conventional MRI findings				0.094
Normal	31 (35.6%)	14 (29.8%)	17 (42.5%)	
VA hypoplasia or stenosis	38 (43.7%)	19 (40.4%)	19 (47.5%)	
Focal stenosis or dilatation	7 (8.0%)	5 (10.6%)	2 (5.0%)	
Pathognomonic findings of dissection (definite dissection)	11 (12.6%)	9 (19.1%)	2 (5.0%)	

Data are presented as mean ± standard deviation or number (%), as appropriate.

Information on prior cervical trauma was available for 64 patients.

HR-VWMRI, High-resolution vessel wall MRI; TFCA, Transfemoral catheter angiography; BMI, Body mass index; HT, Hypertension; DM, Diabetes mellitus; HL, Hyperlipidemia; VA, Vertebral artery.

Among the 87 eligible patients, 47 underwent advanced imaging, either with HR-VWMRI (n = 43) or TFCA (n = 7). Within this group, 40 underwent HR-VWMRI only, 3 underwent TFCA only, and 4 underwent both examinations. In the latter group, inter-modality agreement was perfect (κ = 1.0) (Fig. S3). Patients with advanced imaging were younger and less likely to have hypertension, diabetes, or a history of stroke. Additionally, they were more likely to experience headaches, prior cervical trauma, and limb weakness (Table 1).

**Fig. 2:**
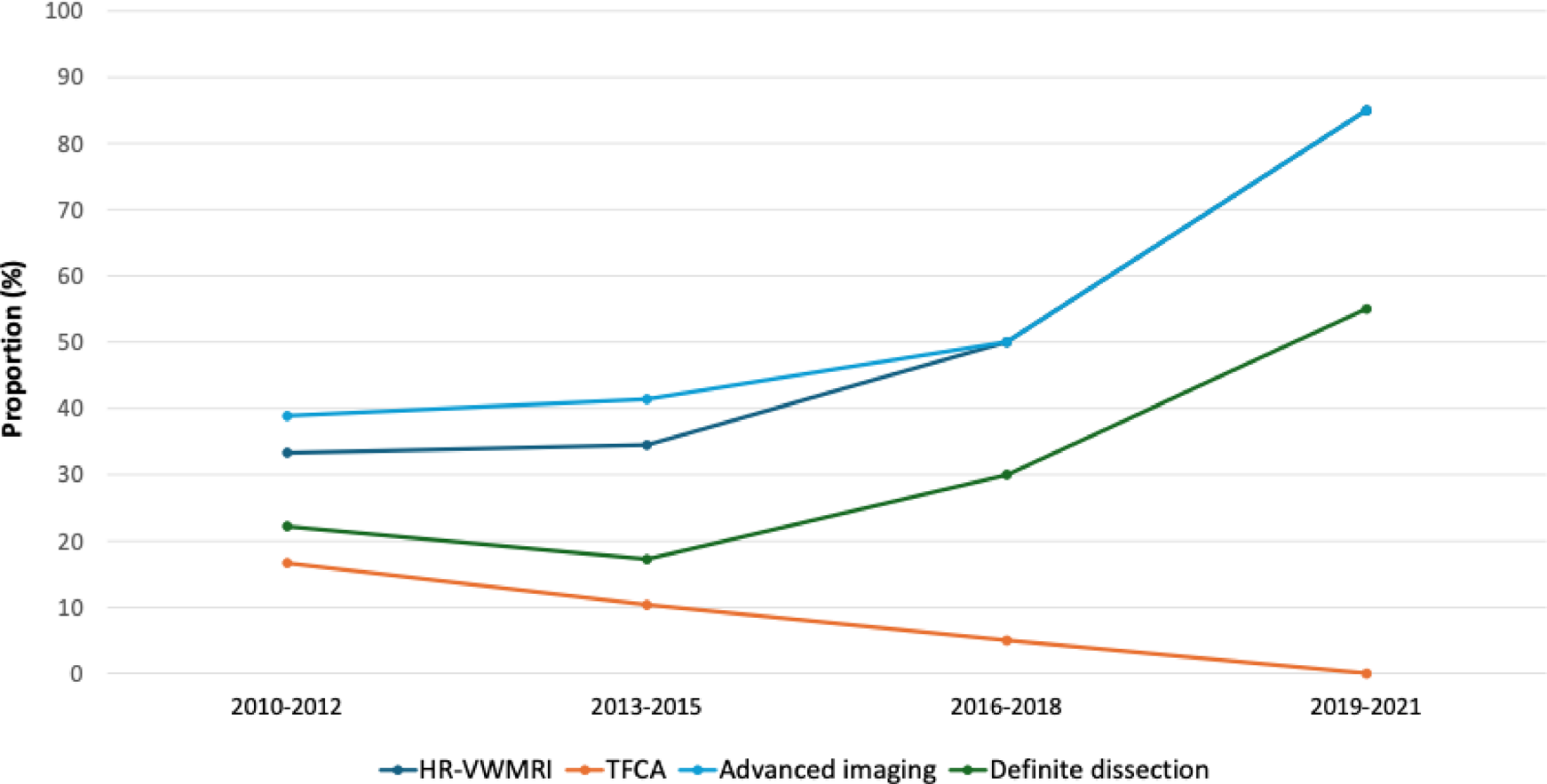
Utilization of advanced imaging modalities and prevalence of definite arterial dissection in LMI over time. Line graph depicting the proportion of patients with LMI who underwent high‑resolution vessel‑wall MRI (HR‑VWMRI; dark blue), transfemoral cerebral angiography (TFCA; orange), or any advanced imaging modality (light blue), alongside the proportion with pathognomonic findings of definite arterial dissection on advanced imaging (green) in successive 3‑year intervals. Over time, the proportion of advanced imaging performed primarily using HR‑VWMRI has increased, and the proportion of definite dissection diagnoses has also risen.

Among those who underwent advanced imaging, 24 (51.1%) had pathognomonic findings of dissection, resulting in a diagnosis of definite dissection (Table 2). Compared to those without dissection, individuals with definite dissection were younger, had a lower body mass index (BMI), and were less likely to have hypertension. They were also more likely to present with headache occurring concurrently with or prior to other neurological symptoms. Over time, the proportion of patients receiving advanced imaging increased, primarily through HR-VWMRI, paralleled by an increasing rate of definite dissection diagnoses (Fig. 2). Univariable logistic regression analysis in this subgroup further indicated that younger age, lower BMI (< 25 kg/m^2^), absence of hypertension, and presence of headaches were significantly associated with definite dissection (Table 3).

**Table 2:** Comparison of characteristics by definite dissection diagnosis in patients with advanced imaging.

	Definite dissection (n = 24)	No dissection (n = 23)	*P*-value
Age	44.5 ± 8.3	60.9 ± 16.4	< 0.001
Sex, male	15 (62.5%)	19 (82.6%)	0.225
BMI	23.9 ± 3.0	25.8 ± 3.4	0.046
HT	8 (33.3%)	17 (73.9%)	0.013
DM	3 (12.5%)	6 (26.1%)	0.286
HL	17 (70.8%)	16 (69.6%)	> 0.99
Smoking	10 (41.7%)	13 (56.5%)	0.468
Stroke history	1 (4.2%)	2 (8.7%)	0.609
Onset situation			> 0.99
During activity	17 (70.8%)	16 (69.6%)	
Wake-up	7 (29.2%)	7 (30.4%)	
Headache			0.002
Simultaneous	5 (20.8%)	3 (13.0%)	
Preceding	15 (62.5%)	5 (21.7%)	
Prior cervical trauma (n = 38)	14 (63.6%)	7 (43.8%)	0.375
Gait disturbance	19 (79.2%)	16 (69.6%)	0.674
Dizziness/vertigo	19 (79.2%)	16 (69.6%)	0.674
Lateropulsion	16 (66.7%)	15 (65.2%)	> 0.99
Sensory abnormality	21 (87.5%)	16 (69.6%)	0.168
Dysarthria	12 (50.0%)	11 (47.8%)	> 0.99
Limb ataxia	12 (50.0%)	8 (34.8%)	0.447
Nausea/vomiting	14 (58.3%)	8 (34.8%)	0.185
Dysphagia	8 (33.3%)	11 (47.8%)	0.475
Facial palsy	8 (33.3%)	7 (30.4%)	> 0.99
Horner syndrome	9 (37.5%)	7 (30.4%)	0.839
Limb weakness	10 (41.7%)	4 (17.4%)	0.134
Hoarseness	3 (12.5%)	6 (26.1%)	0.286
Conventional MRI findings			< 0.001
Normal	3 (12.5%)	11 (47.8%)	
VA hypoplasia or stenosis/occlusion	8 (33.3%)	11 (47.8%)	
Focal stenosis and dilatation	4 (16.7%)	1 (4.3%)	
Pathognomonic findings of dissection	9 (37.5%)	0 (0.0%)	

Data are presented as mean ± standard deviation or number (%), as appropriate.

Information on prior cervical trauma was available in 38 patients.

BMI, Body mass index; HT, Hypertension; DM, Diabetes mellitus; HL, Hyperlipidemia; VA, Vertebral artery.

**Table 3:** Logistic regression analysis for predicting arterial dissection in lateral medullary infarction.

	Univariable logistic regression			Multivariable logistic regression*		
	Coefficient	Odds ratio	*P*-value	Coefficient	Odds ratio	*P*-value
Age ≤ 45 years	1.8971	6.67 (1.56–28.52)	0.011	2.0652	7.89 (0.96–64.48)	0.054
BMI < 25 kg/m^2^	1.1394	3.12 (0.95–10.29)	0.061	3.0851	21.87 (1.49–320.75)	0.024
No hypertension	1.7346	5.67 (1.61–19.97)	0.007	1.6318	5.11 (0.83–31.55)	0.079
Headache						
Simultaneous	1.8326	6.25 (1.03–38.08)	0.047	1.8077	6.10 (0.40–93.05)	0.19
Preceding	2.4204	11.25 (2.52–50.27)	0.002	4.0467	57.21 (3.33–982.67)	0.005

BMI, Body mass index.

*Adjusted for age, BMI, hypertension, simultaneous headache, and preceding headache.

**Fig. 3:**
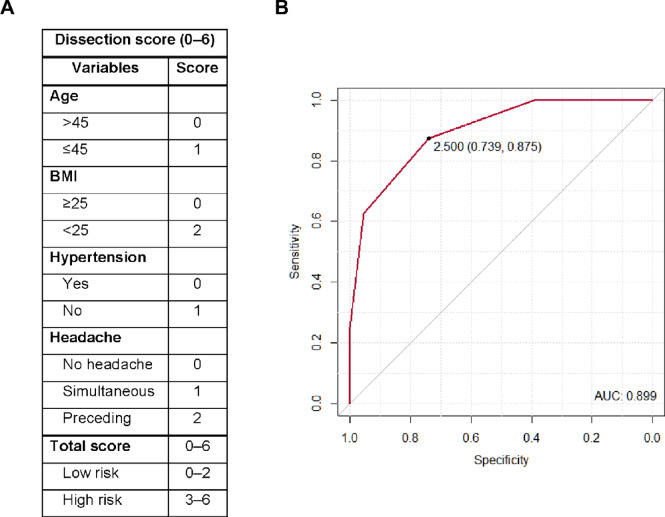
The dissection score and its receiver operating curve (ROC) analysis. (**A**) Dissection score for predicting dissection in patients with lateral medullary infarction based on the multivariable model. (**B**) In the ROC analysis of the dissection score, the optimal cutoff for predicting definite dissection was 2.5, with a sensitivity of 87.5% and specificity of 73.9%. AUC, Area under the curve.

Based on the multivariable logistic regression analysis (Table 3), a dissection score was developed incorporating age, BMI, hypertension, and headache as predictors (Fig. 3A). Each patient’s score was calculated by summing the weighted points assigned to these variables. In the ROC curve analysis, the score demonstrated strong predictive performance for definite dissection, with an area under the curve of 0.899 (95% confidence interval, 0.819–0.980) (Fig. 3B). At the optimal cutoff of 2.5 points, the score yielded a sensitivity of 87.5% (74.3–100.7%), specificity of 73.9% (56.0–91.9%), positive predictive value of 77.8% (62.1–93.5%), and negative predictive value of 85.0% (69.4–100.6%). Patients scoring higher than the cutoff value (≥ 3) were considered having a high dissection score and classified as possible dissection cases.

In the advanced imaging group, patients were stratified by conventional MRI findings and correlated with subsequent HR-VWMRI or TFCA results (Fig. S4). While all patients with pathognomonic findings on conventional MRI showed consistent findings on advanced imaging, 8 (42.1%) of those with VA hypoplasia or stenosis/occlusion, and 4 (80%) of those with focal stenosis or dilatation, which are findings that may be nonspecific, also showed definitive signs of dissection on advanced imaging. Of 15 patients lacking pathognomonic features on conventional MRI but ultimately diagnosed with dissection, 14 (93.3%) had dissection scores exceeding the cutoff value (Table 4). This included three patients later confirmed to have PICA dissection despite unremarkable initial imaging.

**Table 4:** Case summary of lateral medullary infarction patients without pathognomonic findings on conventional MRI but diagnosed with definite dissection through advanced imaging.

No	Sex/Age	Stroke risk factors	Headache	Prior cervical trauma	Other clinical manifestations	Findings on conventional MRI	Findings on advanced imaging	Dissection score
1	F/54	HL	Preceding headache	No	Sensory abnormality, dizziness/vertigo, gait disturbance, lateropulsion, nausea/vomiting, Horner syndrome, limb ataxia	Unremarkable	Intramural hematoma, PICA	5
2	F/53	HL, smoking (current smoker)	Simultaneous headache	No	Dysarthria, dizziness/vertigo, gait disturbance, lateropulsion, nausea/vomiting, limb ataxia	Unremarkable	Intraluminal flap, PICA	4
3	M/47	HT, HL, smoking (ex-smoker)	Preceding headache	Yes	Sensory abnormality, limb weakness, dizziness/vertigo, gait disturbance, lateropulsion, facial palsy	Unremarkable	Intraluminal flap, PICA	4
4	M/49	HL, smoking (ex-smoker)	Preceding headache	Yes	Sensory abnormality, limb weakness, dizziness/vertigo, gait disturbance, lateropulsion, nausea/vomiting, Horner syndrome	Hypoplastic VA and distal occlusion	Intramural hematoma, VA	3
5	M/47	HL, smoking (current smoker)	Simultaneous headache	Yes	Sensory abnormality, dysarthria, dizziness/vertigo, gait disturbance, lateropulsion, limb ataxia	Hypoplastic VA	Dissecting aneurysm, VA	4
6	F/45	HL	Preceding headache	Unknown	Sensory abnormality, limb weakness, dysatrheia, hoarseness, dysphagia, gait disturbance, lateropulsion, diplopia	VA occlusion	Tapering occlusion of VA in TFCA	4
7	M/36	Smoking (ex-smoker)	Preceding headache	Unknown	Sensory abnormality, gait disturbance, lateropulsion, limb ataxia	Hypoplastic VA and distal occlusion	Intramural hematoma, VA	4
8	F/30	HL	Preceding headache	Yes	Dizziness/vertigo, gait disturbance, lateropulsion, nausea/vomiting	Hypoplastic VA and distal occlusion	Intramural hematoma, VA	6
9	F/32	DM, HL	Preceding headache	Yes	Sensory abnormality, dysarthria, dizziness/vertigo, hoarseness, dysphagia, gait disturbance, lateropulsion, nausea/vomiting, Horner syndrome, facial palsy	Hypoplastic VA and distal stenosis	Intramural hematoma, VA	4
10	M/56	HT, DM	Preceding headache	Yes	Sensory abnormality, limb weakness, dysarthria, dizziness/vertigo, gait disturbance, nausea/vomiting, facial palsy, limb ataxia	Distal VA occlusion	Intramural hematoma, VA	2
11	F/34	HL	No headache	Yes	Sensory abnormality, dizziness/vertigo, gait disturbance, lateropulsion	Distal VA stenosis	Intraluminal flap, intramural hematoma, VA	4
12	M/53	None	Preceding headache	Unknown	Sensory abnormality, dysarthria, dizziness, hoarseness, dysphagia, gait disturbance, lateropulsion, nausea/vomiting, nystagmus	Focal stenosis and dilatation, VA	Intramural hematoma, VA	3
13	F/39	None	Simultaneous headache	Unknown	Sensory abnormality, dizziness, gait disturbance, lateropulsion, nausea/vomiting, nystagmus, diplopia, facial palsy	Focal stenosis and dilatation, VA	Double lumen appearance, VA	5
14	F/32	None	Preceding headache	Unknown	Sensory abnormality, limb weakness, dysarthria	Focal stenosis and dilatation, VA	Tapering diffuse stenosis of VA in TFCA	6
15	M/43	HT, HL, smoking (current smoker)	No headache	No	Sensory abnormality, dizziness	Focal stenosis and dilatation, PICA	Intramural hematoma, PICA	3

HT, Hypertension; DM, Diabetes mellitus; HL, Hyperlipidemia; VA, Vertebral artery; PICA, Posterior inferior cerebellar artery.

**Fig. 4:**
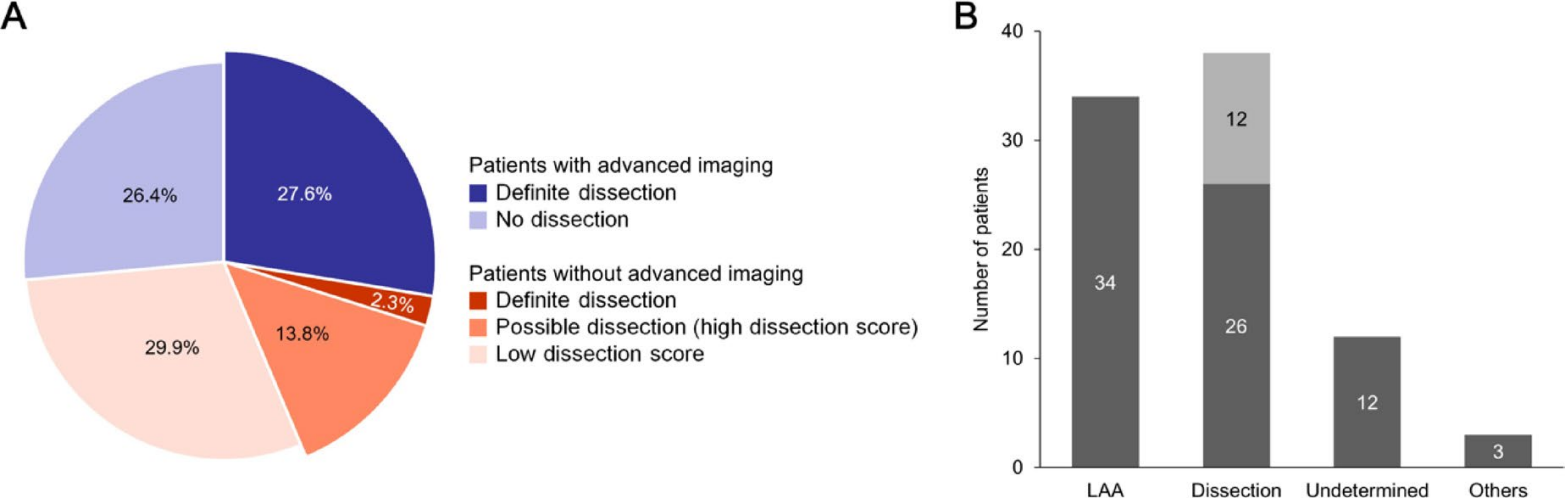
The prevalence of definite and possible dissection among patients with lateral medullary infarction. (**A**) The distribution of definite, possible, and no dissection in patients with or without advanced imaging. The combined prevalence of definite and possible dissection accounted for 43.7%. (**B**) Number of patients according to stroke etiology, including possible dissection identified with dissection score. Twelve patients without advanced imaging nor pathognomonic findings of dissection on conventional MRI were classified as possible dissection (light grey). LAA, Large artery atherosclerosis.

The score was also applied to patients without advanced imaging. In addition to two cases with pathognomonic findings on conventional MRI, 12 of 38 patients (31.6%) had dissection scores above the cutoff and were classified as possible dissection cases (Fig. 4A). The characteristics of possible dissection cases are summarized in Table S1. Among these patients, five underwent follow-up conventional MRI, and resolution of the initial vascular abnormalities was observed in two cases. When definite and possible dissection cases were combined, arterial dissection accounted for 43.7% of LMI cases, suggesting it may represent a potentially predominant etiology in this stroke subtype (Fig. 4B).

## Discussion

In this study, 51.1% of LMI patients who underwent advanced imaging were diagnosed with definite arterial dissection. Definite dissection was associated with younger age, absence of hypertension, lower BMI, and presence of headache. Based on these factors, the dissection score was proposed as an exemplary risk stratification tool. The score effectively predicted dissection in those who lacked pathognomonic findings on conventional MRI but were later confirmed to have dissection by advanced imaging. When applied to those without advanced imaging, the score identified additional possible dissection cases. Overall, definite and possible dissections accounted for 43.7% of LMI cases, exceeding the proportion attributed to large artery atherosclerosis.

Previous studies have rarely emphasized arterial dissection in LMI, with most reporting a prevalence below 30% based solely on conventional imaging. Even the most recent studies showed a prevalence below 10%^8,12^, and only one study that incorporated TFCA data reported a prevalence above 30%^10^. However, while TFCA is limited by its invasiveness and associated procedural risks, HR-VWMRI allows non-invasive evaluation of both the vessel wall and lumen, offering superior diagnostic performance^6,17^. As such, prevalence estimates of dissection based on HR-VWMRI are likely to be more reliable.

In this study, 91.5% of patients with advanced imaging underwent HR-VWMRI. Conventional MRI demonstrated a low sensitivity for dissection (37.5%), whereas HR-VWMRI detected all cases of PICA dissection missed by conventional imaging. Moreover, the increased utilization of HR-VWMRI over time paralleled a rise in definite dissection diagnoses. These findings suggest that previous studies may have substantially underestimated the true prevalence of dissection.

Although HR-VWMRI offers clear diagnostic benefits, its use is limited by reliance on clinician suspicion, cost, and availability. This highlights the need for a simple early risk stratification tool to identify patients most likely to benefit from advanced imaging. The dissection score proposed in this study serves as an exemplary model for developing an early risk stratification tool, by systematically integrating multiple predictors and their relative weights rather than relying solely on subjective clinical judgment. Notably, although trauma history has traditionally been regarded as a hallmark of dissection, it was not significantly associated with dissection in this cohort. This suggests that spontaneous dissections may predominate in LMI^18^, and demonstrates the risk of relying on individual predictors.

Among patients classified as having possible dissection, some cases demonstrated resolution of initial vascular abnormalities in their follow-up images (Table S1). Early utilization of a suitable risk stratification tool may help minimize diagnostic delays in such patients, which is crucial given that dissection has a distinct risk–benefit profile for extended antithrombotic therapy^19^. Furthermore, if it is sufficiently validated and appropriately adapted to the relevant population, such a tool may assist in making a provisional diagnosis in resource-limited settings where advanced imaging is not readily available.

In this study, BMI was inversely associated with dissection and contributed substantially to the dissection score. This aligns with findings from large-scale studies on cervical artery dissection^20,21^ and a Mendelian randomization analysis^22^, which have shown that genetically higher BMI is linked to a lower risk of cervical arterial dissection. Proposed mechanisms include different connective tissue composition and increased arterial wall stiffness associated with higher BMI. However, for vertebral artery dissection, studies have failed to demonstrate its consistent association with BMI, possibly due to the lower incidence of vertebral dissection cases^22,23^. Moreover, those studies were mainly conducted in Western populations. By replicating the inverse relationship between BMI and dissection in a clinically well-defined cohort of LMI in an Asian population, the present study supports the existence of a fundamental biological mechanism of dissection that may transcend ethnicity, cultural background, and the specific cervical artery involved.

This study has several limitations. First, its single-center retrospective design and small sample size may have introduced selection bias. Patients judged at higher risk for dissection were more likely to undergo advanced imaging, which should be considered when interpreting the dissection score. Although the high rate (> 80%) of advanced imaging in the recent subset may mitigate some bias, studies applying advanced imaging to all LMI patients are warranted. Second, inclusion of only East Asian patients limits generalizability to other ethnic groups. Moreover, excluding those with lesions outside the lateral medulla may also limit generalizability. However, by focusing on isolated cases at lateral medulla, which is supplied by PICA or VA perforators, the study targeted a well-recognized and clinically distinct syndrome likely caused by local vascular disease, enhancing clinical relevance and applicability. Future studies expanding to stroke at PICA or VA territory would provide further insights. Third, the validation of the score was limited. Obtaining advanced imaging data for possible dissection cases would have aided validation, but this was not feasible due to the observational design and risk of false negatives from dissection resolution. Hence, only limited follow-up information for possible dissection cases was provided. Prospective studies applying advanced imaging to all LMI patients, as well as multi-center studies are warranted.

In conclusion, this study suggests that, with advanced imaging data, arterial dissection may be more common in LMI than previously recognized. This highlights the need to raise awareness of dissection in LMI and to implement active, resource-efficient diagnostic evaluations based on risk stratification. The proposed scoring system serves as an example of a potential risk stratification tool, but its utilization and interpretation should consider the study’s limitations. Prospective and multi-center studies are warranted to further validate these findings.

## Electronic supplementary material

Below is the link to the electronic supplementary material.

Supplementary Material 1

## Data Availability

Data supporting the findings of this study are available from the corresponding author upon reasonable request.
