# Supplementary material for: Re-evaluation of arterial dissection as a possible major cause of lateral medullary infarction in a single-center study from South Korea
Source: Sci Rep. 2025 Jul 19;15:26272. doi: 10.1038/s41598-025-11860-8 (PMC12276257; doi:10.1038/s41598-025-11860-8)
Supplement: Supplementary file 1 — Supplementary Material 1 [file 41598_2025_11860_MOESM1_ESM.pdf]

Manuscript title: Re-evaluation of arterial dissection as a possible major cause of lateral medullary infarction in a single-center study from South Korea

Author list: Jiyeon Ha, MD; Wookjin Yang, MD, PhD ; Eung-Joon Lee, MD; Han-Yeong Jeong, MD; Matthew Chung, MD; Hyemin Jang, MD, PhD; Jeong-Min Kim, MD, PhD; Keun-Hwa Jung, MD, PhD; Seung-Hoon Lee, MD, PhD

Supplementary Tables

Table S1. Case summary of possible dissection cases

Supplementary Figures

Figure S1. Classification of findings on conventional MRA in pure LMI

Figure S2. Representative figure of pathognomonic findings of dissection on high-resolution vessel wall MRI (HR-VWMRI)

Figure S3. Venn diagram of advanced imaging and dissection status

Figure S4. Distribution of advanced imaging findings in relation to conventional MRI results

Table S1. Case summary of possible dissection cases

No.	Sex/Age	Stroke risk factors	Headache	BMI	Prior cervical trauma	Findings on conventional MRI	Findings on follow up imaging	mRS at 3 months	Dissection score
1	M/72	HT, HL, Smoking	Simultaneous headache	< 25	No	Normal	VA stenosis	1	3
2	M/52	HT, DM, HL, Smoking	Simultaneous headache	< 25	Unknown	Hypoplastic VA and distal stenosis	Not available	1	3
3	M/56	HT, DM, HL	Simultaneous headache	< 25	No	Normal	Normal	1	3
4	M/45	HT	No headache	< 25	No	Hypoplastic VA	Not available	0	3
5	M/70	HT, DM	Simultaneous headache	< 25	No	Hypoplastic VA and distal occlusion	Hypoplastic VA and distal occlusion	2	3
6	M/33	None	Preceding headache	< 25	Yes	Hypoplastic VA and distal stenosis	Normal	0	6
7	M/53	HL	No headache	< 25	Unknown	Normal	Not available	1	3
8	F/80	HT, DM	Simultaneous headache	< 25	Unknown	Normal	Not available	1	3
9	F/49	None	No headache	< 25	No	Distal VA stenosis and PICA nonvisualization	Normal	2	3
10	M/64	HL, Smoking	No headache	< 25	Unknown	Normal	Not available	1	3
11	M/78	HT, Smoking	Preceding headache	< 25	Unknown	Hypoplastic VA	Not available	4	4
12	F/71	DM	No headache	< 25	No	Normal	Not available	1	3

HT, hypertension; DM, diabetes mellitus; HL, hyperlipidemia; VA, vertebral artery; PICA, posterior inferior cerebellar artery.

Supplementary figures and legends

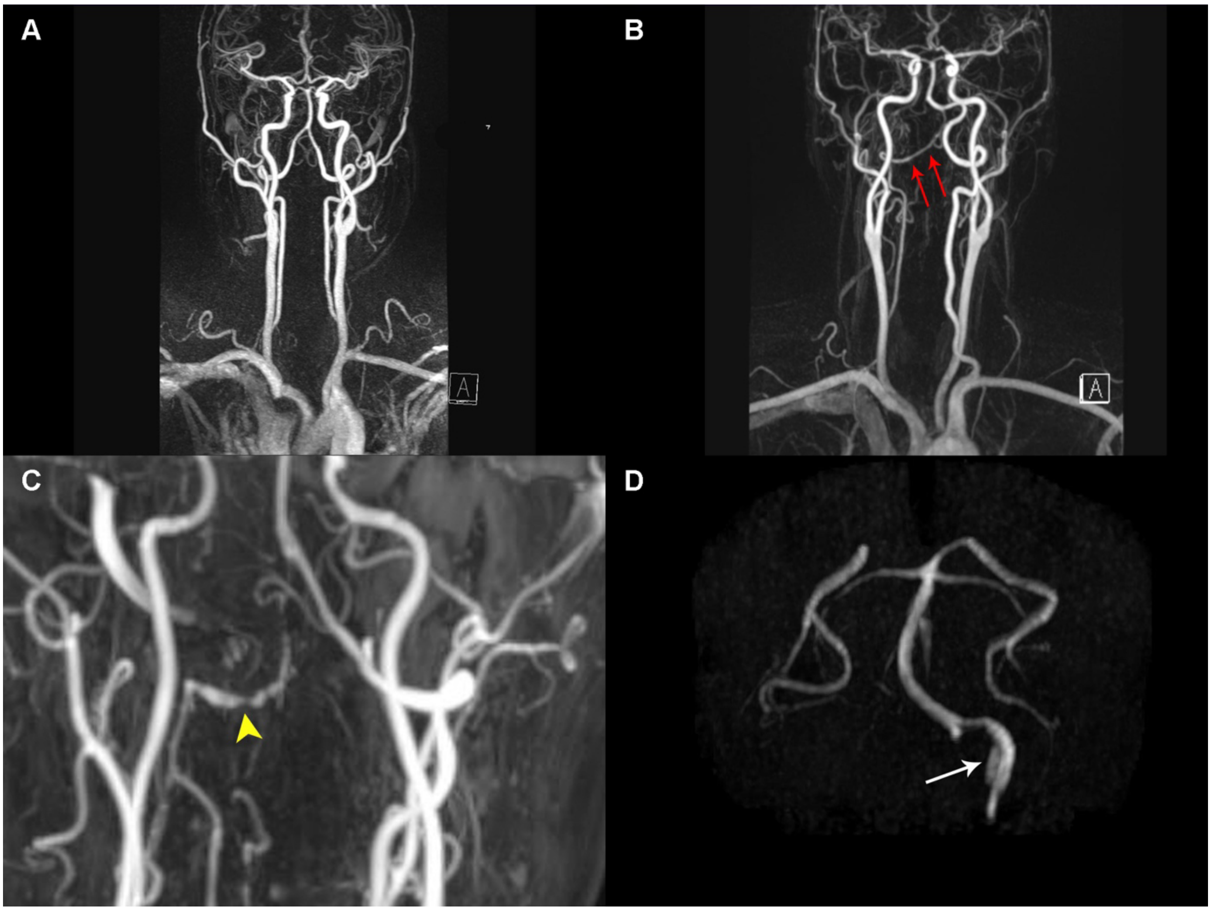

Figure S1. Classification of findings on conventional MRA in pure LMI

The findings of vertebral artery (VA) or posterior inferior cerebellar artery ipsilateral to the infarction site in each patient were categorized as follows: **(A)** Normal, **(B)** VA stenosis, occlusion, or hypoplasia (red arrows indicate right VA hypoplasia), **(C)** focal stenosis and dilation (pearl-and-string sign) indicated by yellow arrowhead, and **(D)** pathognomonic findings of dissection, including dissecting aneurysm (white arrow).

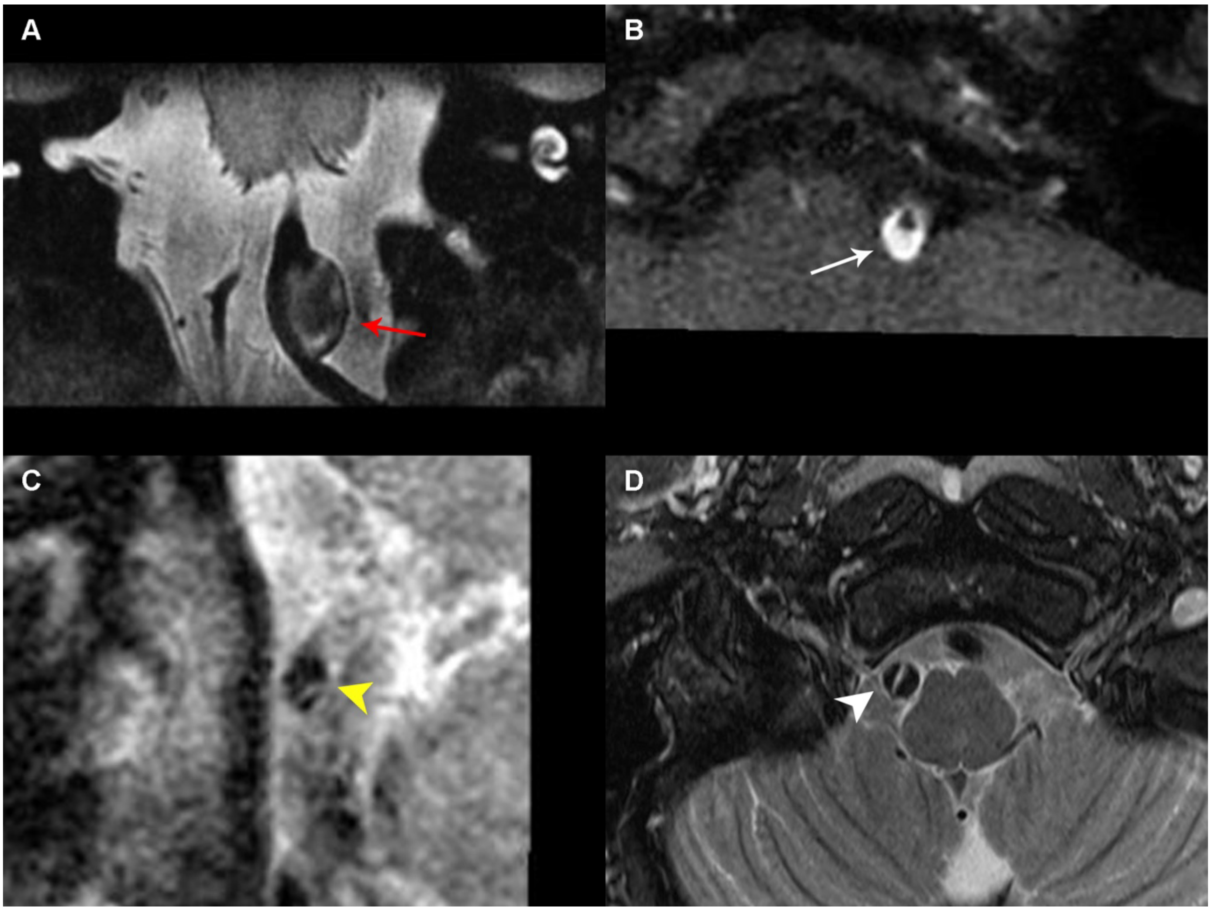

Figure S2. Representative figure of pathognomonic findings of dissection on high-resolution vessel wall MRI (HR-VWMRI)

Examples of the four pathognomonic features of arterial dissection on HR-VWMRI. **(A)** Dissecting aneurysm (red arrow) on T2-weighted imaging (T2WI), **(B)** intramural hematoma (white arrow) on T1-weighted imaging with gadolinium enhancement, **(C)** intraluminal flap (yellow arrowhead) on T2WI, and **(D)** double-lumen appearance (white arrowhead) on T2WI.

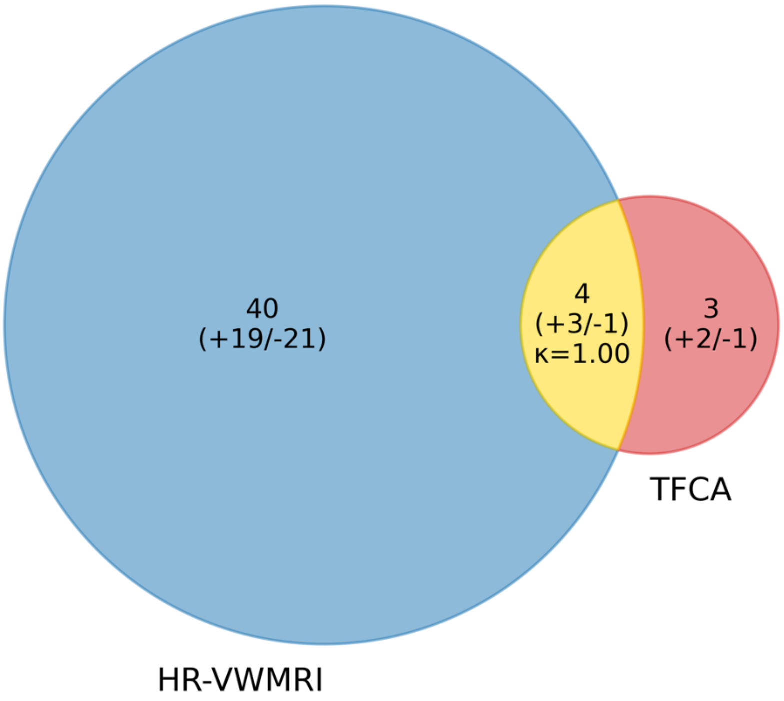

Figure S3. Venn diagram of advanced imaging and dissection status

Venn diagram summarizing the forty-seven patients who received high-resolution vessel-wall MRI (HR-VWMRI) and/or transfemoral cer[1]ebral angiography (TFCA). HR-VWMRI alone was performed in forty patients, TFCA alone in three, and both modalities in four. In the group that underwent both examinations the two methods yielded identical dissection results, giving a kappa value of 1.0. Numbers inside each segment show the total examined with that modality combination, followed by the counts classified as dissection-positive (+) and dissection-negative (−).

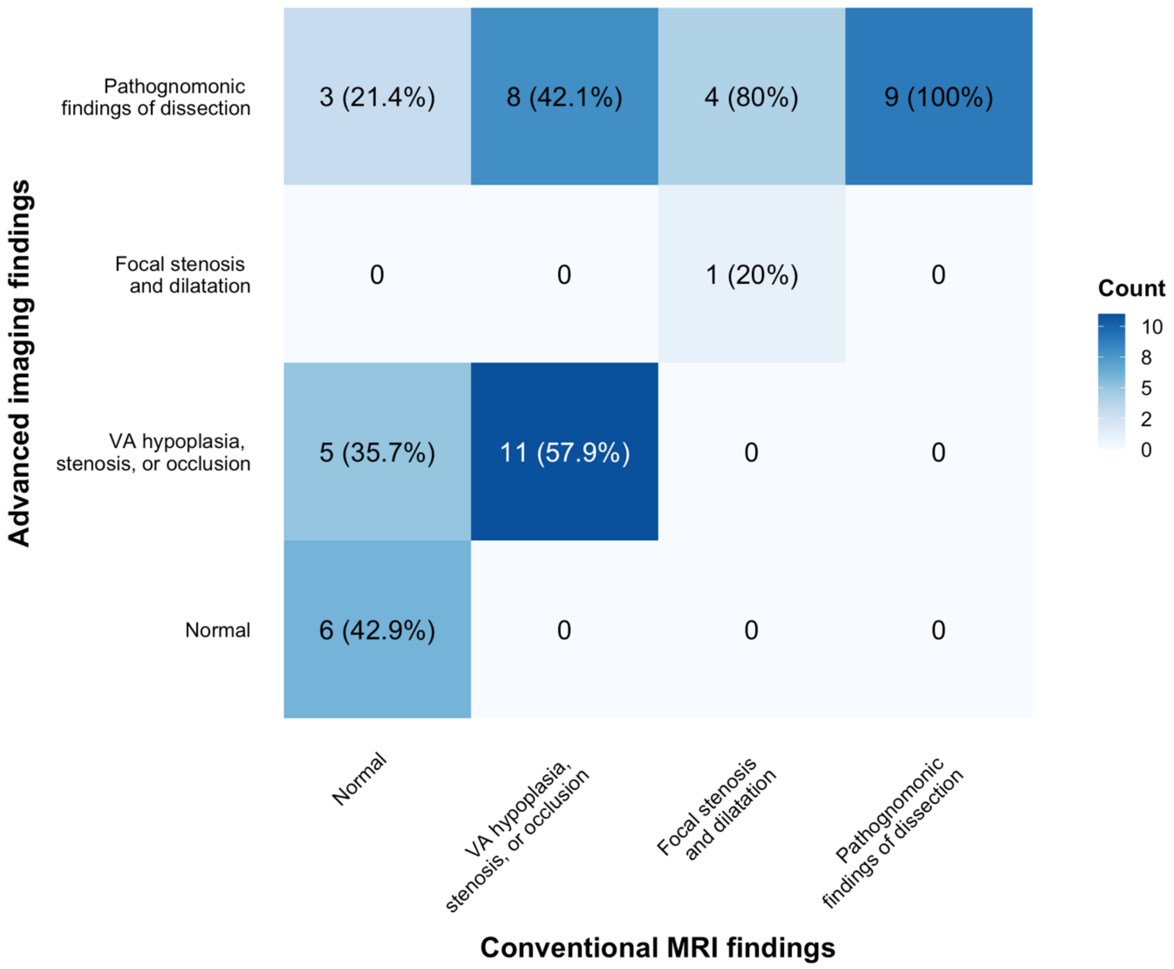

Figure S4. Distribution of advanced imaging findings in relation to conventional MRI results

Patients who underwent advanced imaging were categorized according to their conventional MRI findings, and the results of their advanced imaging were analyzed accordingly. Percentages were calculated within each conventional MRI finding category. Although pathognomonic findings for dissection identified by conventional MRI were highly specific, there were 15 false-negative cases of definite dissection. VA, vertebral artery.
